# Supplementary material for: SHB1 and CCA1 interaction desensitizes light responses and enhances thermomorphogenesis
Source: Nat Commun. 2019 Jul 15;10:3110. doi: 10.1038/s41467-019-11071-6 (PMC6629618; doi:10.1038/s41467-019-11071-6)
Supplement: Supplementary file 1 — Supplementary Information [file 41467_2019_11071_MOESM1_ESM.pdf]

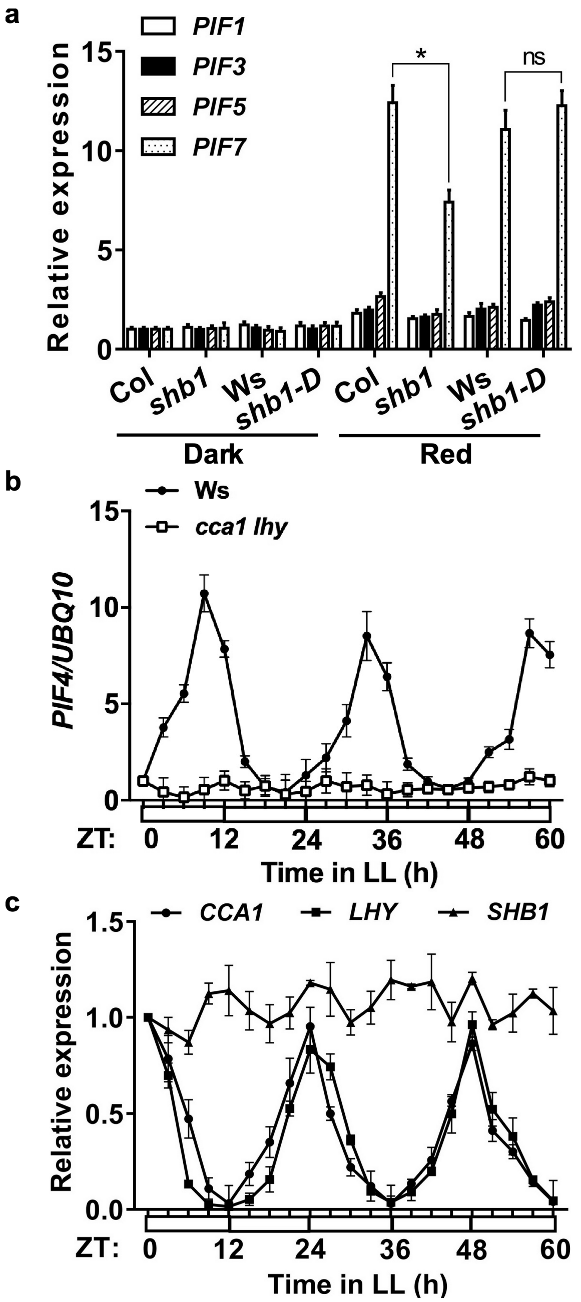

Supplementary Fig. 1 SHB1 does not upregulate the expression of a few other *PIFs*. (a) *PIF1, PIF3, PIF5 and PIF7* expression in Col, *shb1*, Ws, and *shb1-D*. Seedlings were grown in dark for 4 days and then remained in the dark or transferred to 15 μmol m^{-2} s^{-1} red light for 3 hours. (b) *PIF4* expression in Ws and *cca1 lhy* under continuous white light (LL) after entrained under 12-hr light and 12-hr dark for 7 days from two biological replicates. (c) *CCA1*, *LHY* and *SHB1* expression in Ws seedlings that were entrained under 12-hr light and 12-hr dark for 7 days, released into continuous white light (LL), and sampled every 3 hrs for 60 hrs. Primers used for *SHB1* are SHB1 F and SHB1 R. Gene expression in each sample was normalized to that of *UBQ10*, and data are presented as the means±SE calculated from two biological replicates. Source data are provided as a Source Data file.

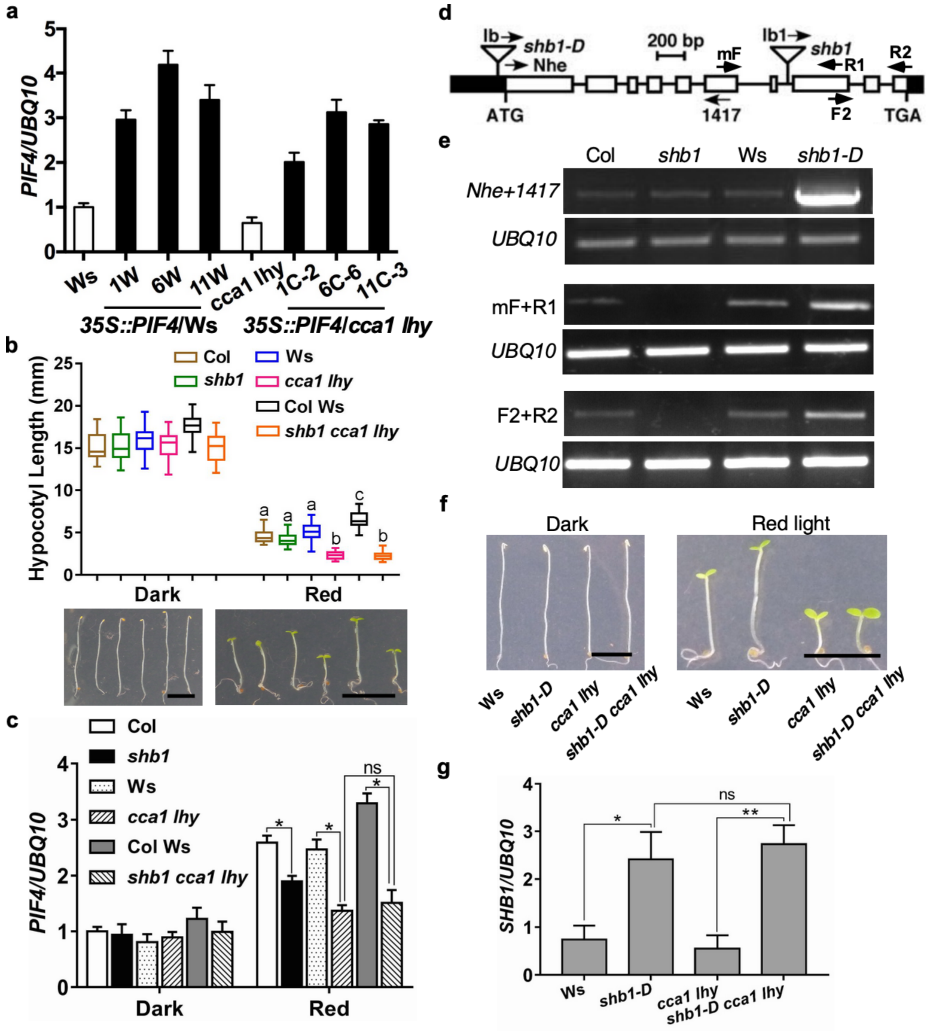

Supplementary Fig. 2 *PIF4* is overexpressed in either Ws or *cca1 lhy*. (a) *PIF4* expression driven by the CaMV 35S promoter in each line was normalized to that of *UBQ10*, and data are presented as the means±SE calculated from two biological replicates. Seedlings were grown in a growth room with a 16L/8D cycle at 22 °C under 30 μmol m^{-2} s^{-1} fluorescent white light for 4 days. Hypocotyl lengths (b) of and *PIF4* expression (c) in Col, *shb1*, Ws, *cca1 lhy,* Col Ws, and *shb1 cca1 lhy* in the dark or under 10 μmol m^{-2} s^{-1} red light for 4 days. Several Col Ws lines were derived from the same cross when each *shb1*, *cca1* or *lhy* allele was genotyped and showed consistent phenotypes. One representative line was shown. Box plots display medians as horizontal lines, interquartile ranges as boxes and whiskers extending 1.5 times the interquartile range. (d) *SHB1* sequence coordination from BAC clone T30C3. Black boxes represent the untranslated regions. White boxes indicate exons, and lines indicate introns. (e) *SHB1* and *UBQ10* expression in Col, *shb1*, Ws and *shb1-D* for 35 cycles using Nhe and 1417, mF and R1, F2 and R2 or *UBQ10* primer pair. (f) Hypocotyl images of Ws, *shb1-D*, *cca1 lhy* and *shb1-D cca1 lhy* in the dark and under 10 μmol m^{-2} s^{-1} red light for 4 days. Bar=5 mm. (g) *SHB1* and *UBQ10* expression in Ws, *shb1-D*, *cca1 lhy* and *shb1-D cca1 lhy* using SHB1 F and SHB1 R as in Supplementary Fig. 1c or *UBQ10* primer pair. For e and g, seedlings were grown as in a. Source data are provided as a Source Data file.

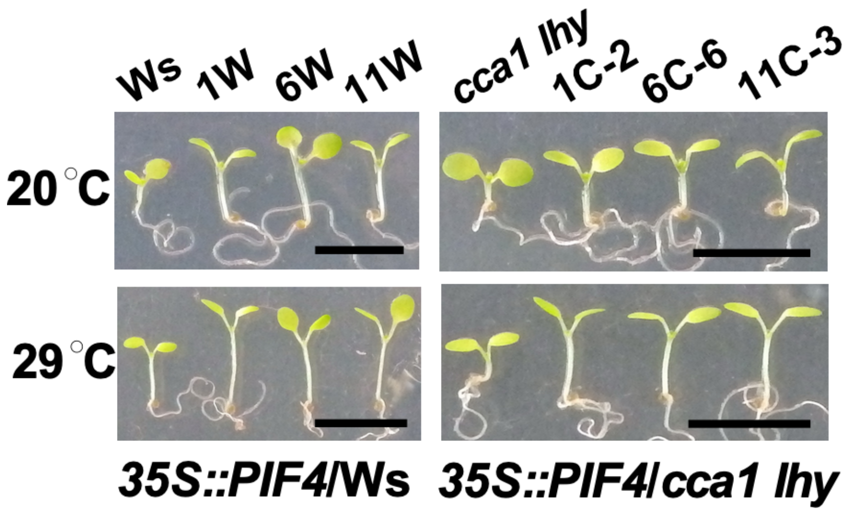

Supplementary Fig. 3 Hypocotyl images of *35S::PIF4:GFP* lines in Ws or *cca1 lhy* grown under 30 µmol m^{-2} s^{-1} white light at 20 °C for 7 days or 20 °C for 4 days followed by 29 °C for 3 days. Bar=5 mm.

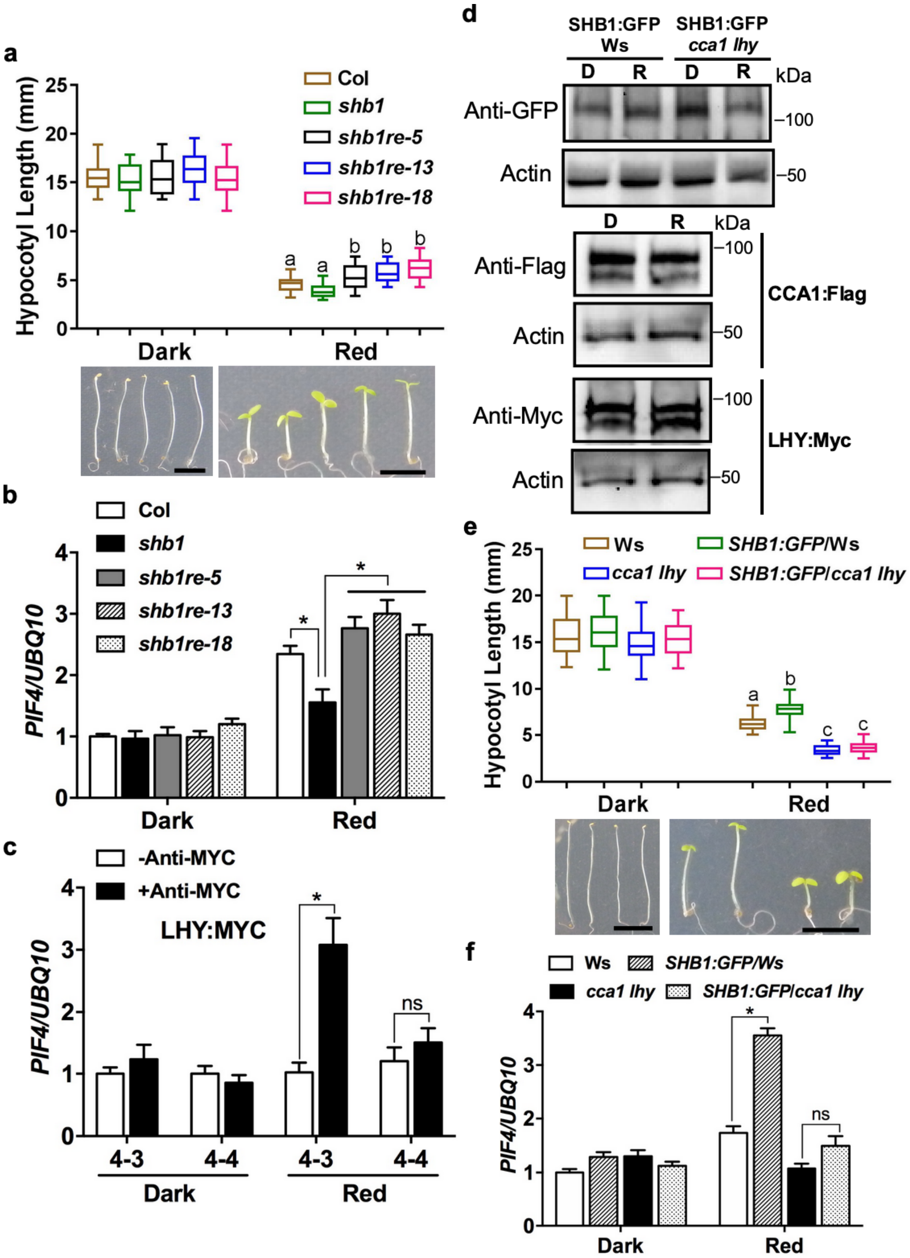

Supplementary Fig. 4 SHB1:GFP fusion protein is functional. Hypocotyl lengths (a) of and *PIF4* expression (b) in Col, *shb1* and three transgenic plants that carry *35S::SHB1:GFP* in *shb1*. Box plots display medians as horizontal lines, interquartile ranges as boxes and whiskers extending 1.5 times the interquartile range. (c) Association of LHY:MYC with the *PIF4* promoter. Seedlings were grown in the dark or under 15 μmol m^{-2} s^{-1} red light for 5 days. (d) Accumulation of SHB1:GFP in Ws and *cca1 lhy* as well as CCA1:FLAG and LHY:MYC in the dark and under red light. Actin was probed as loading controls. Hypocotyl lengths (e) of and *PIF4* expression (f) in transgenic plants that carry *35S::SHB1:GFP* in Ws and *cca1 lhy*. Gene expression or enrichment of DNA fragments was normalized to that of *UBQ10*. Data are presented as the means±SE calculated from three biological replicates. Bar=5 mm. Source data are provided as a Source Data file.

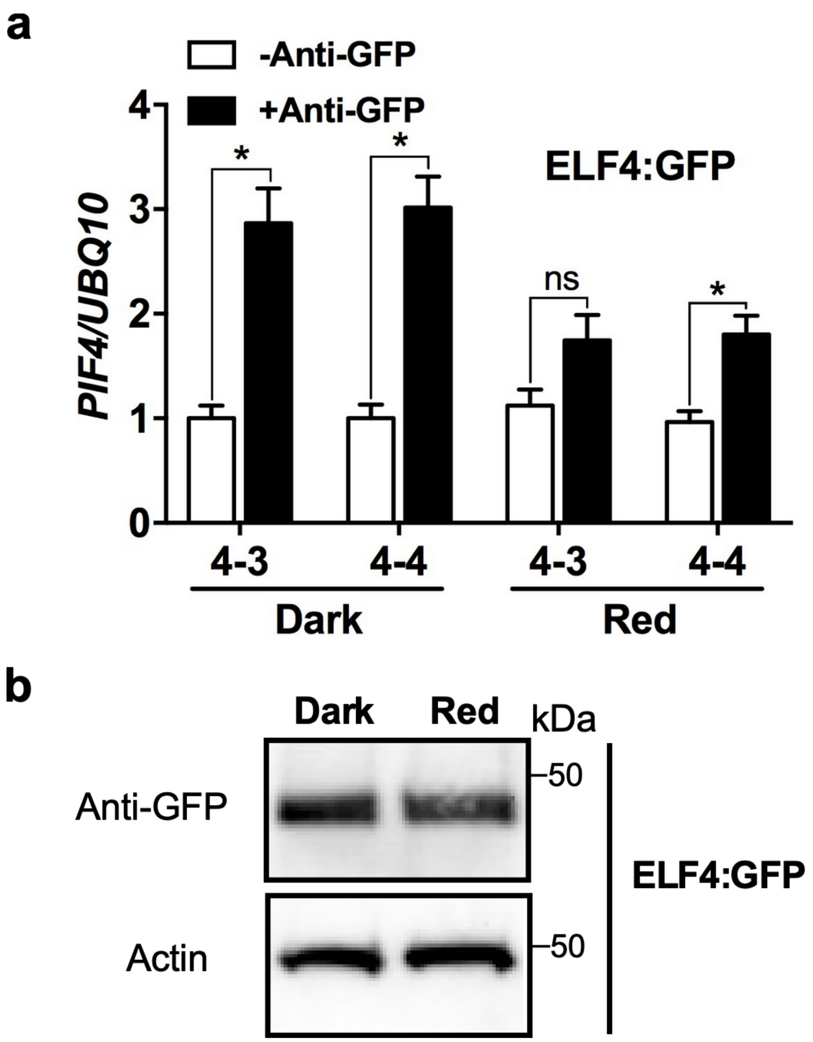

Supplementary Fig. 5 LUX or NOX and CCA1 or LHY have nearby binding sites in the *PIF4* promoter. (a) Association of ELF4:GFP with the *PIF4* promoter in the dark and under red light in two biological replicates. (b) Accumulation of ELF4:GFP in the dark or under 15 μmol m^{-2} s^{-1} red light for 5 days in two biological replicates. Actin was probed as loading controls. Source data are provided as a Source Data file.

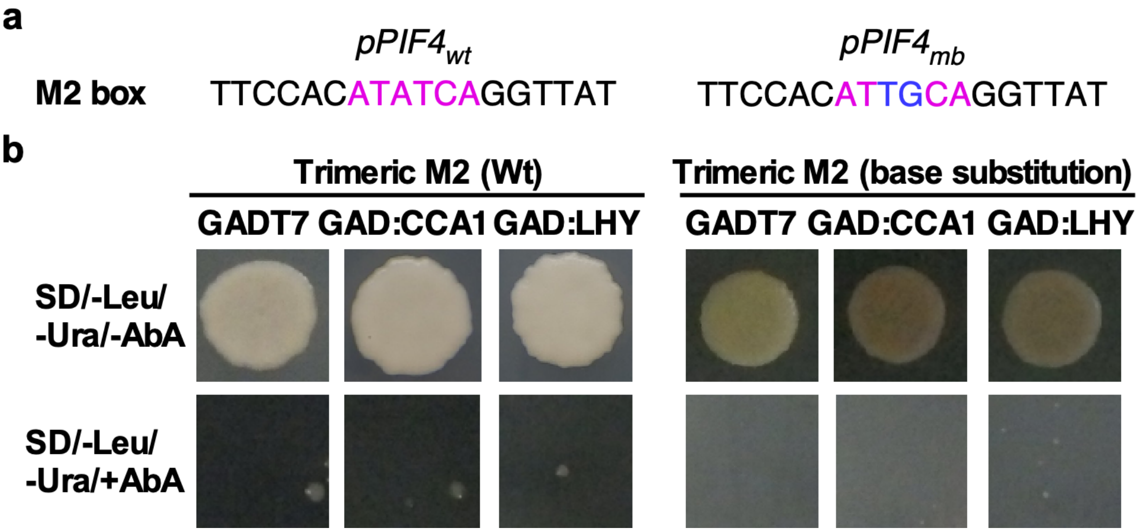

Supplementary Fig. 6 CCA1 and LHY do not bind Myb box M2 in the *PIF4* promoter. (a) Core sequences of the potential MYB binding element M2 are noted in red and the bases mutated by base substitution (mb) are noted in blue. (b) Y1H assays for CCA1 or LHY in pGADT7 over trimeric repeats of wild type (left) or mutated (right) M2 element. Each repeat has a 6-bp flanking sequence on either side of the M2 core element. Golden Y1H cells were grown on SD/-Leu/-Ura media with or without 300 ng/ml Aureobasidin A (AbA) selection.

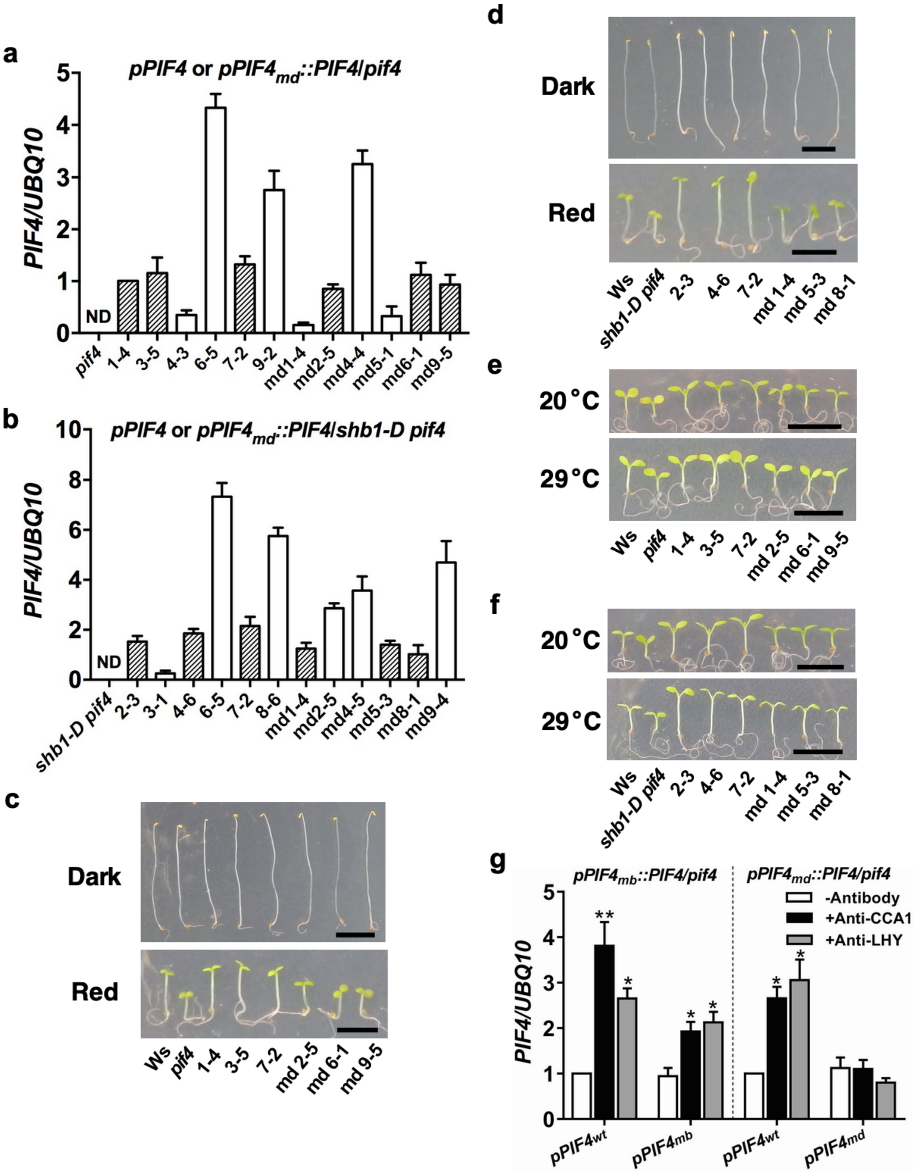

Supplementary Fig. 7 *pPIF4* or *pPIF4md::PIF4* transgene are introduced to *pif4* or *shb1-D pif4*. *PIF4* expression in *pif4* (a) or *shb1-D pif4* (b) lines under 50 μmol m^{-2} s^{-1} white light normalized to that of *UBQ10* in one biological replicate and *PIF4* expression in 1-4 was set at 1. The shaded lines were selected for further hypocotyl and *PIF4* expression analysis. Hypocotyl images of Ws, *pif4* and *pPIF4* or *pPIF4md*::*PIF4* in *pif4* (c) and Ws, *shb1-D pif4* and *pPIF4* or *pPIF4md*::*PIF4* in *shb1-D pif4* (d) grown in the dark or under 10 μmol m^{-2} s^{-1} red light for 4 days. Hypocotyl images of Ws, *pif4* and *pPIF4* or *pPIF4md*::*PIF4* in *pif4* (e) and Ws, *shb1-D pif4* and *pPIF4* or *pPIF4md*::*PIF4* in *shb1-D pif4* (f) under 30 μmol m^{-2} s^{-1} white light at 20 °C for 7 days or 20 °C for 4 days followed by 29 °C for 3 days. Bar=5 mm. (g) Association of CCA1 and LHY with the *pPIF4* and *pPIF4mb* or *pPIF4md* promoter in 4-day-old dark-grown seedlings exposed to 15 μmol m^{-2} s^{-1} red light for 3 hr from two biological replicates. A forward primer that contained a wild type, base-substituted or deleted M1 box was used with a common reverse primer in qPCR analysis. Enrichment of DNA fragments was quantified by qPCR and normalized to that of *UBQ10*. Source data are provided as a Source Data file.

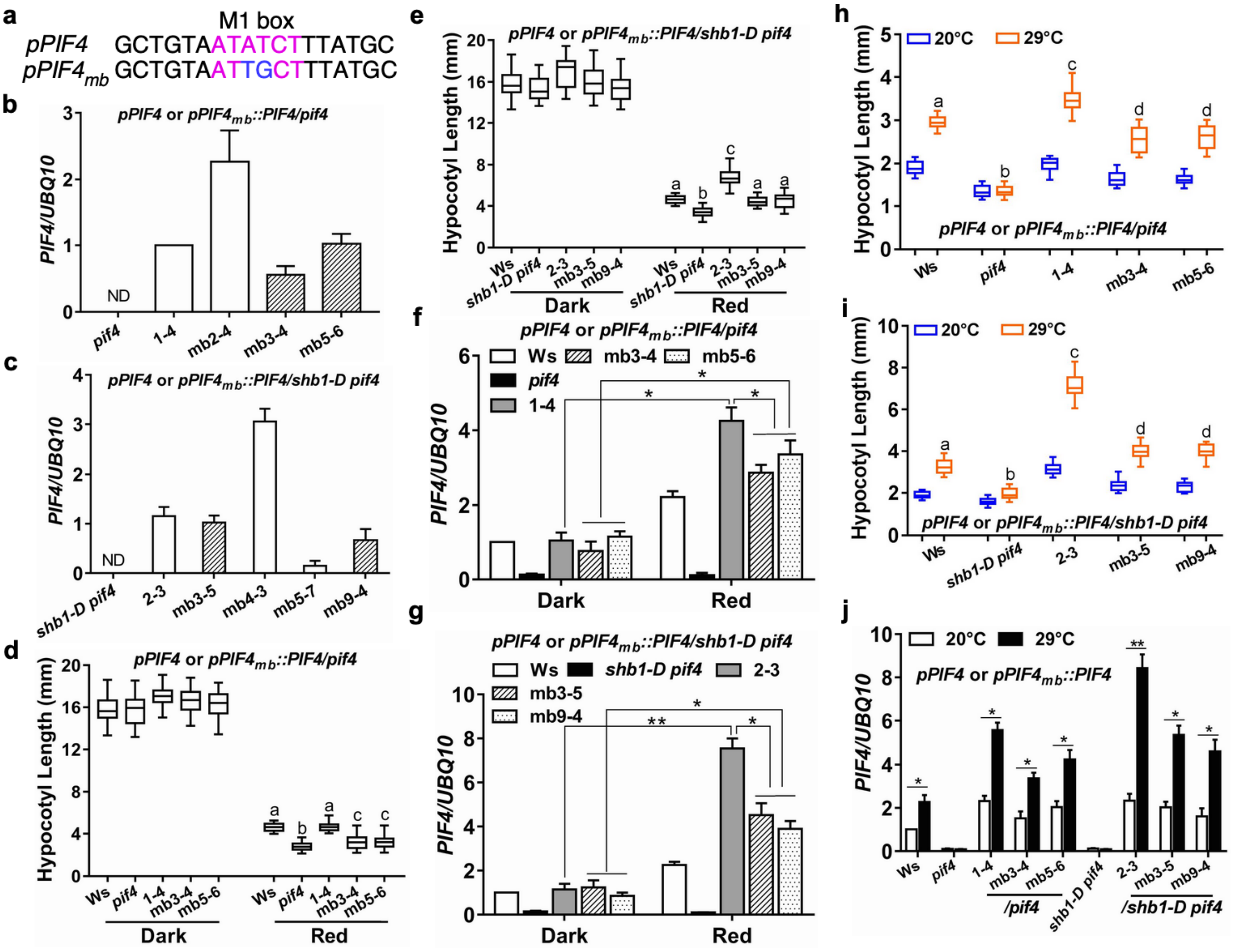

Supplementary Fig. 8 *pPIF4mb::PIF4* transgene is introduced to *pif4* or *shb1-D pif4*. (a) MYB binding element M1 is noted in red, and the bases mutated are noted in blue. *PIF4* expression in *pif4* (b) or *shb1-D pif4* (c) lines under 50 µmol m^{-2} s^{-1} white light normalized to that of *UBQ10* in one biological replicate and *PIF4* expression in 1-4 was set at 1. The shaded lines were selected for further hypocotyl and *PIF4* expression analysis. Hypocotyl lengths of Ws, *pif4* and *pPIF4* or *pPIF4mb*::*PIF4* in *pif4* (d) and Ws, *shb1-D pif4* and *pPIF4* or *pPIF4mb*::*PIF4* in *shb1-D pif4* (e). *PIF4* expression in Ws, *pif4* and *pPIF4* or *pPIF4mb*::*PIF4* in *pif4* (f) and Ws, *shb1-D pif4* and *pPIF4* or *pPIF4mb*::*PIF4* in *shb1-D pif4* (g) from two biological replicates. The seedlings were grown in the dark or under 10 µmol m^{-2} s^{-1} red light for 4 days. Hypocotyl lengths of Ws, *pif4* and *pPIF4* or *pPIF4mb*::*PIF4* in *pif4* (h) and Ws, *shb1-D pif4* and *pPIF4* or *pPIF4mb*::*PIF4* in *shb1-D pif4* (i) under 30 µmol m^{-2} s^{-1} white light at 20 °C for 7 days or 20 °C for 4 days followed by 29 °C for 3 days. *PIF4* expression in Ws, *pif4*, *pPIF4* or *pPIF4mb*::*PIF4* in *pif4*, *shb1-D pif4* and *pPIF4* or *pPIF4mb*::*PIF4* in *shb1-D pif4* (j) grown at 20 °C for 5 days and then incubated at 20 °C or 29 °C for 4 h from two biological replicates. Significance levels by Student's two-tailed heteroscedastic t tests in d: $p<0.001$ between a and b or a and c, $p<0.05$ between b and c; in e: $p<0.001$ between a and b, a and c or b and c; in h: $p<0.001$ between a and b, a and c, b and c or c and d, $p<0.01$ between a and d; in i: $p<0.001$ between a and b, a and c, b and c, b and d or c and d; $p<0.01$ between a and d. Source data are provided as a Source Data file.

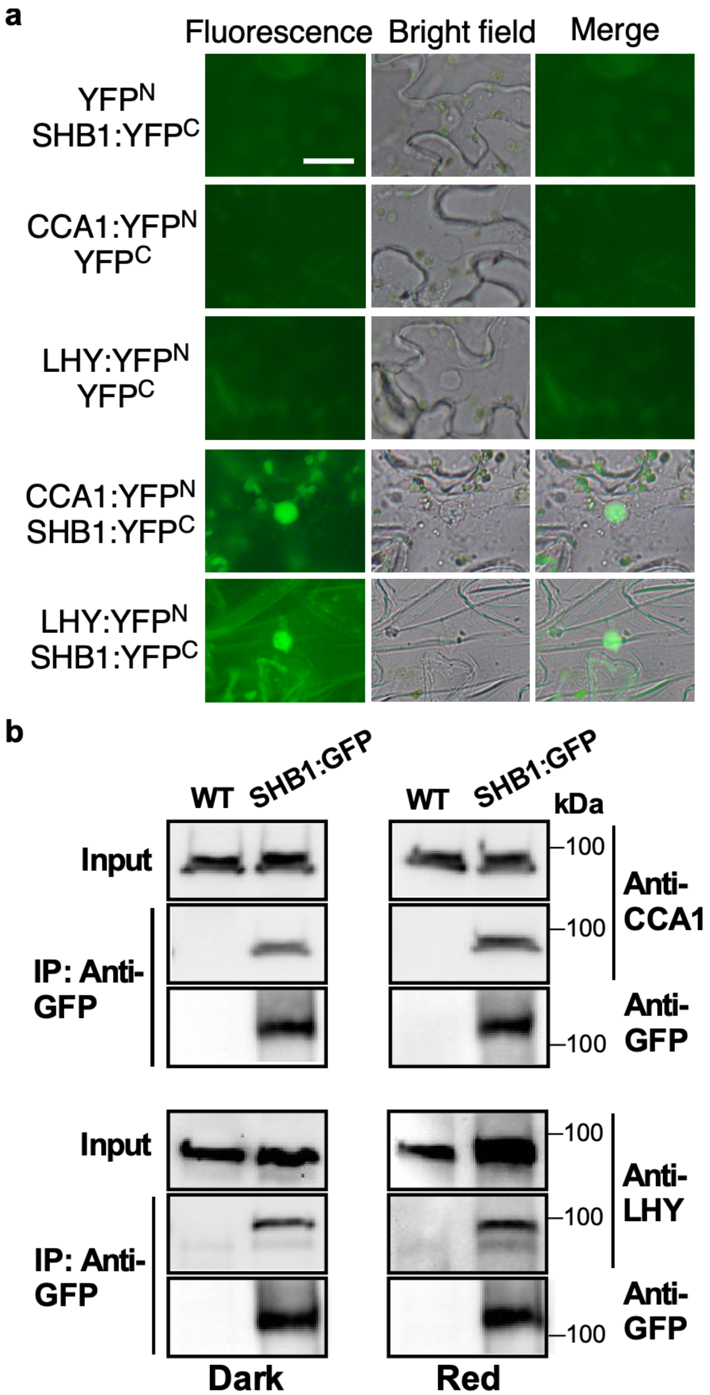

Supplementary Fig. 9 SHB1 interacts with CCA1 and LHY in the dark or under red light. (a) BiFC assays of YFP^N with SHB1:YFP^C, CCA1:YFP^N or LHY:YFP^N with YFP^C, and CCA1:YFP^N or LHY:YFP^N with SHB1:YFP^C in *Nicotiana* leaf epidermal cells in the dark or under red light. Fluorescent images were captured 48 to 72 hours after Agrobacteria transfection. Bar=10 μm. (b) Co-immunoprecipitation (Co-IP) of CCA1 or LHY by SHB1:GFP in protein extracts prepared from transgenic *Arabidopsis* and immuno-precipitated with anti-GFP antibody. Protein blots were probed with anti-CCA1 or anti-LHY antibodies. Source data are provided as a Source Data file.

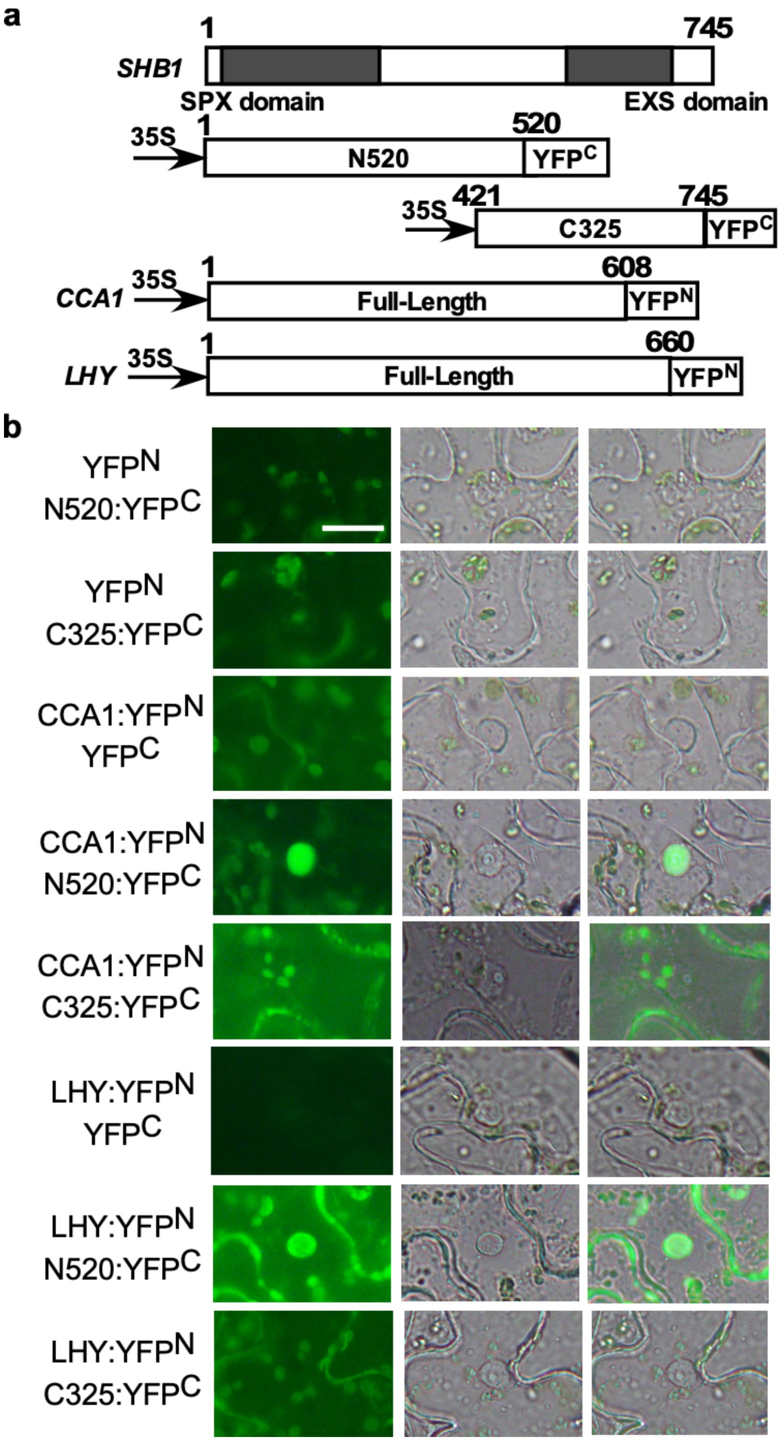

Supplementary Fig. 10 SHB1 N-terminus interacts with CCA1 or LHY. (a) Full-length and various deletion derivatives of SHB1 were fused with YFP^C at their C termini. Full-length CCA1 or LHY was fused with YFP^N at their C termini. All constructs were driven by the CaMV 35S promoter. The numbers above each construct indicate amino acid sequence coordination. (b) BiFC assays of YFP^C fusion of SHB1 N-terminus or C-terminus with full-length CCA1:YFP^N or LHY:YFP^N in *Nicotiana* leaf epidermal cells. Fluorescent images were captured 48 to 72 hours after Agrobacteria transfection. Bar=10 µm.

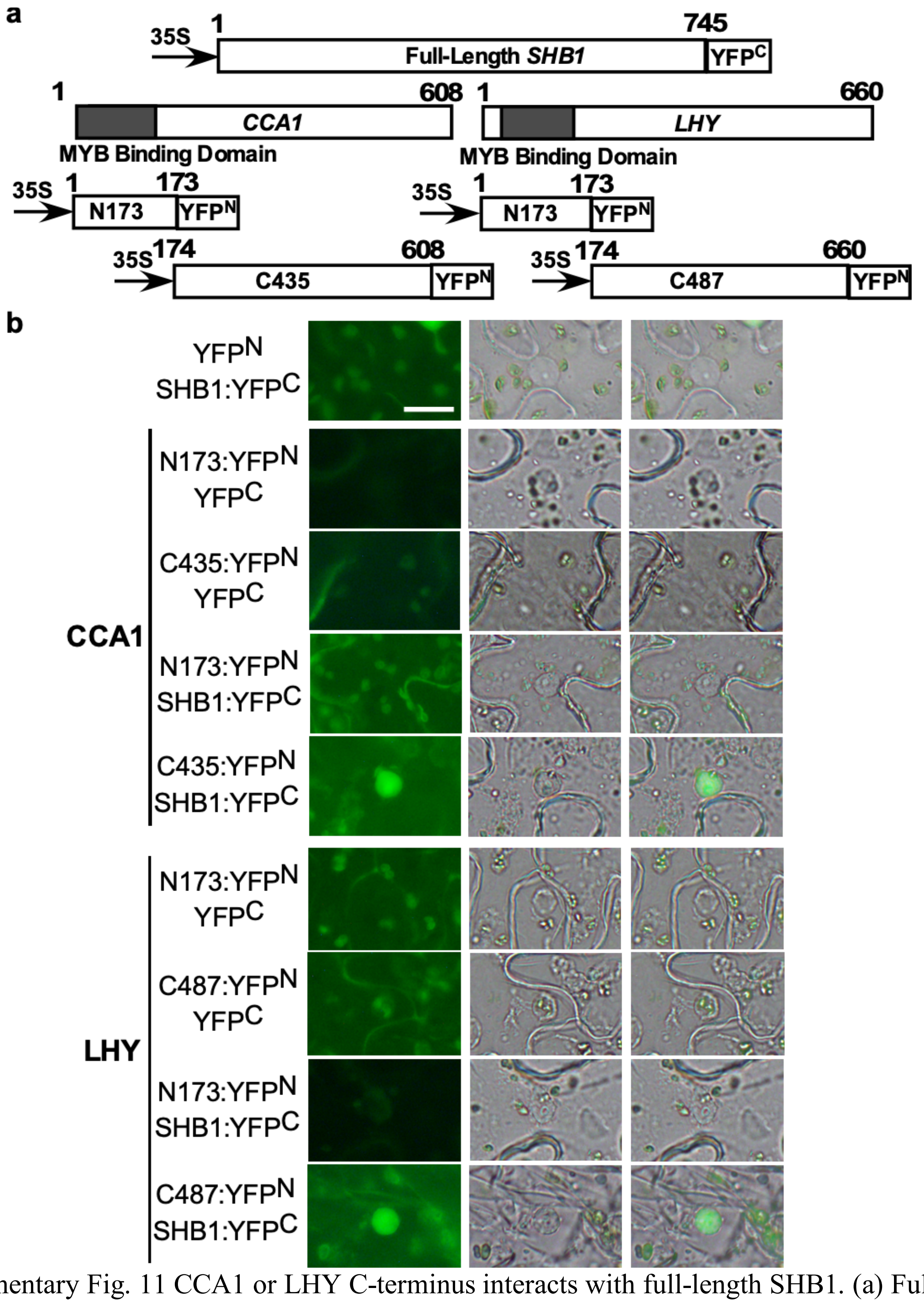

Supplementary Fig. 11 CCA1 or LHY C-terminus interacts with full-length SHB1. (a) Full-length and various deletion derivatives of CCA1 or LHY were fused with YFP^{N} at their C termini. Full-length SHB1 was fused with YFP^{C} at its C termini. All constructs were driven by the CaMV 35S promoter. The numbers above each construct indicate amino acid sequence coordination. (b) BiFC assay of YFP^{N} fusion of CCA1 or LHY N-terminus or C-terminus with full-length SHB1:YFP^{C} in *Nicotiana* leaf epidermal cells. Fluorescent images were captured 48 to 72 hours after Agrobacteria transfection. Bar=10 μm.

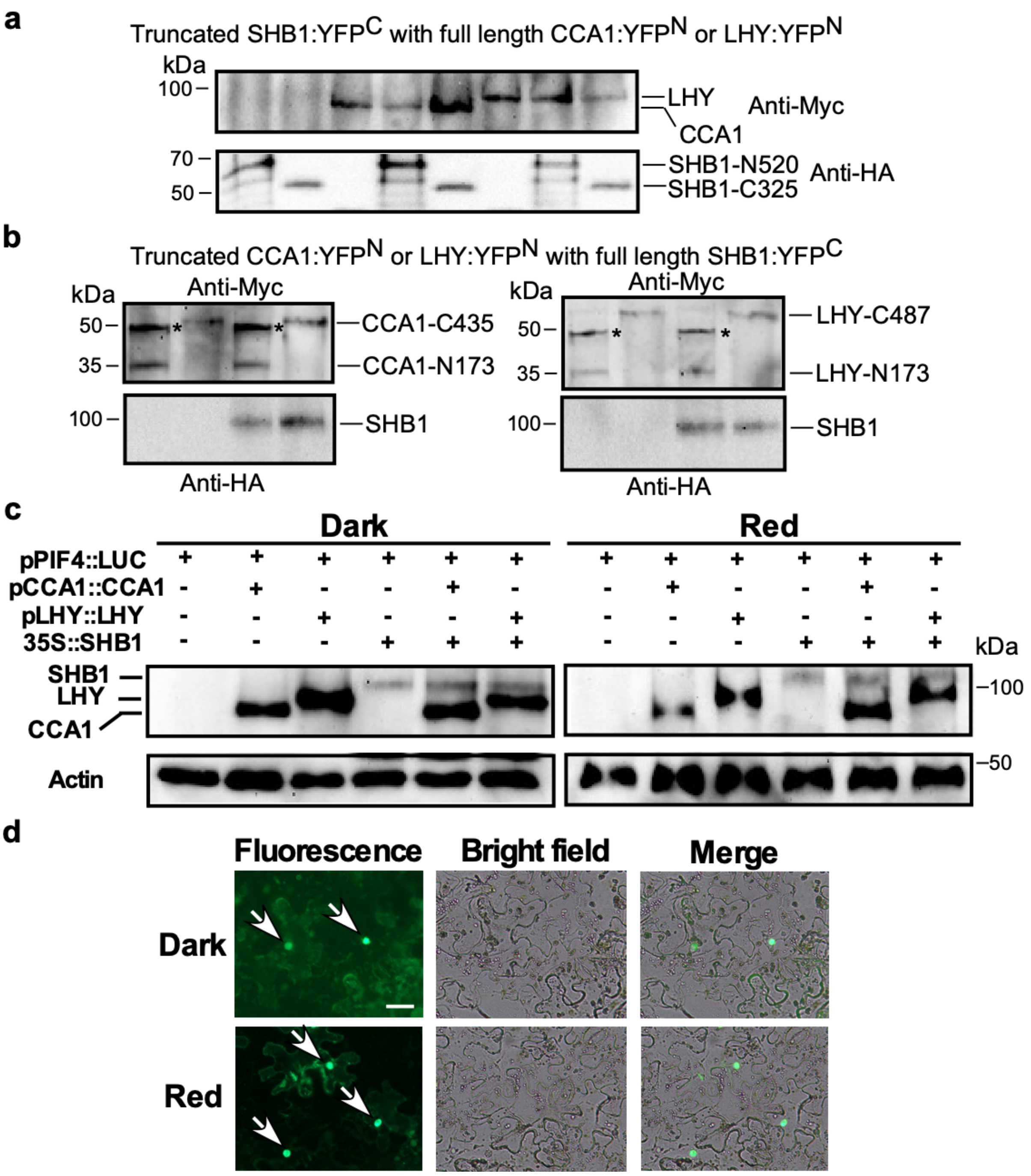

Supplementary Fig. 12 Protein accumulation in domain mapping and transactivation assays. (a) Accumulation of SHB1 N520:YFP^{C} or C325:YFP^{C}, CCA1:YFP^{N} and LHY:YFP^{N} in *Nicotiana* leaves. (b) Accumulation of SHB1:YFP^{C}, CCA1 N173:YFP^{N} or C435:YFP^{N}, and LHY N173:YFP^{N} or C487:YFP^{N} in *Nicotiana* leaves. * indicates a nonspecific band associated with either the CCA1 N173 or LHY N173 construct. (c) Accumulation of CCA1:MYC, LHY:MYC and SHB1:MYC in Arabidopsis leaf protoplasts in the dark and under red light. Source data are provided as a Source Data file. (d) Subcellular localization of SHB1:GFP in *Nicotiana* leaf epidermal cells in the dark and under red light. Bar=5 μm.

Supplementary Table 1 List of primers used in this study

Primer Name	Primer Sequence (5'-3')	Purpose
**Quantitative real-time PCR**		
PIF1 F	AGAAGCCACCACTACTGATGA	Real-time PCR primer for *PIF1*
PIF1 R	TGAAGGAAGGAGGAGGAATAGG	Real-time PCR primer for *PIF1*
PIF3 F	CTGAAAGGAGACGGCGTGATAG	Real-time PCR primer for *PIF3*
PIF3 R	CAGATAGTAACCAGACGCCATTGAC	Real-time PCR primer for *PIF3*
PIF4 F	CTTCAAGTGATGTGGATGGGGA	Real-time PCR primer for *PIF4*
PIF4 R	TGGGAACCCACCGATGTATCT	Real-time PCR primer for *PIF4*
PIF5 F	CAGATAAAGCTTCGATATTGGATGA	Real-time PCR primer for *PIF5*
PIF5 R	TAGCGAGCTGCTCCGATAAGAT	Real-time PCR primer for *PIF5*
PIF7 F	TTGAAACAGCTACAAGCACAAGTACAG	Real-time PCR primer for *PIF7*
PIF7 R	GATTCGAAGAACTTGAAGGCATG	Real-time PCR primer for *PIF7*
SHB1 F	AAGAATGGTGGAAGACAGAGAT	Real-time PCR primer for *SHB1*
SHB1 R	AGAAGCAGCAACGATGGT	Real-time PCR primer for *SHB1*
CCA1 F	GATCTGGTTATTAAGACTCGGAAGCCATATAC	Real-time PCR primer for *CCA1*
CCA1 R	GCCTCTTTCTCTACCTTGGAGA	Real-time PCR primer for *CCA1*
LHY F	AAGTCTCCGAAGAGGGTC	Real-time PCR primer for *LHY*
LHY R	ATGTTCCAACACCGATC	Real-time PCR primer for *LHY*
Nhe	AGCATCACTCAAAACCCAAAAG	Semi-quantitative PCR for *SHB1*
1417	TATAACCAAGTTCAGTGCC	Semi-quantitative PCR for *SHB1*
mF	GGCACTGAACTTGGTTATA	Semi-quantitative PCR for *SHB1*
R1	TCTCTGTCTTCCACCATTCTTC	Semi-quantitative PCR for *SHB1*
F2	CAAGCTTCTTGTTCCTCACAAA	Semi-quantitative PCR for *SHB1*
R2	ATCTTCTTCGTAGTTGAACGGT	Semi-quantitative PCR for *SHB1*

UBQ10 RT/F	TCCAGGACAAGGAGGTATTCCTCCG	Real-time PCR primer for *UBQ10*
UBQ10 RT/R	CCACCAAAGTTTTACATGAAACGAA	Real-time PCR primer for *UBQ10*
cMyc-R	GATCTTCTTCAGAGATCAGTTTCTG	Real-time PCR primer for exogenous *PIF4*
**ChIP-qPCR**		
PIF4-1 F	ATTTTAAAACCACATTGCTAGAGAA	ChIP-PCR primer for *PIF4-1*
PIF4-1 R	CTCACCTAATAATGTTTTTTTTGGT	ChIP-PCR primer for *PIF4-1*
PIF4-2 F	TTGTATTTCTTATTAAGCCAAGGGT	ChIP-PCR primer for *PIF4-2*
PIF4-2 R	CTATAGCGTTATGGTTTTTGTCGAT	ChIP-PCR primer for *PIF4-2*
PIF4-3 F	CAAAAACACCCTCAAATCATTGT	ChIP-PCR primer for *PIF4-3*
PIF4-3 R	AGAAGAAGAAGATGAGAGAGGTTCA	ChIP-PCR primer for *PIF4-3*
PIF4-4 F	AGATAGAGAGTTGTGTTGGGCGT	ChIP-PCR primer for *PIF4-4*
PIF4-4 R	TGATTAAGAGATACAAGGAGGAGCA	ChIP-PCR primer for *PIF4-4*
M1 F	AAAAGATTGCAGTACTGTTGTCCCT	ChIP-PCR primer for *PIF4-M1*
M1 R	CAACTCACAAAACATGAAAGAAAC	ChIP-PCR primer for *PIF4-M1*
M2 F	TGTTTCTTTCATGTTTTGTGAGTTG	ChIP-PCR primer for *PIF4-M2*
M2 R	GAAAGCTTCTGGACGAATCAGAA	ChIP-PCR primer for *PIF4-M2*
UBQ10 F	TCCAGGACAAGGAGGTATTCCTCCG	ChIP-PCR primer for *UBQ10*
UBQ10 R	CCACCAAAGTTTTACATGAAACGAA	ChIP-PCR primer for *UBQ10*
M1$_{wt}$ F	TCCCTCGCTGTA**ATATCT**TTA	ChIP-PCR primers for *PIF4* promoter without or with M1 box base substitution or deletion
M1$_{mb}$ F	TATCTCCCTCGCTGTA**ATTG**	
M1$_{md}$ F	TATCTTCCCTCGCTGTATTA	
M1 R2	GCCCAACACAACTCTCTATCTCT	

**Yeast one-hybrid**		
PIF4 FSal I	gcgtcgacATGTCCCAGAACTTGCCAC	Cloning primer for *PIF4* promoter
PIF4 RXho I	ccctcgagATATTCTGGCAATCTAGGAAAGCTT	Cloning primer for *PIF4* promoter
4M1LBS FKpn I	ggggtaccAATCATTATCTCCCTCGCTGT	Cloning primer for *PIF4* promoter
4M1LBS RXho I	ccctcgagAAGCTTCTGGACGAATCAGAA	Cloning primer for *PIF4* promoter
M1tri F	agcttCTGTAATATCTTTATGCGCTGTAATATCTTTATGCGCTGTAATATCTTTATGg	Cloning oligo for *PIF4* promoter
M1tri R	tcgacCATAAAGATATTACAGCGCATAAAGATATTACAGCGCATAAAGATATTACAGa	Cloning oligo for *PIF4* promoter
M2tri F	agcttTCCACATATCAGGTTATTTCCACATATCAGGTTATTTCCACATATCAGGTTAg	Cloning oligo for *PIF4* promoter
M2tri R	tcgacTAACCTGATATGTGGAAATAACCTGATATGTGGAAATAACCTGATATGTGGAa	Cloning oligo for *PIF4* promoter
M1tri Fmu	agcttCTGTAATTGCTTTATGCGCTGTAATTGCTTATGCGCTGTAATTGCTTTATGCGg	Cloning oligo for mutated *PIF4* promoter
M1tri Rmu	tcgacCGCATAAAGCAATTACAGCGCATAAGCAATTACAGCGCATAAAGCAATTACAGa	Cloning oligo for mutated *PIF4* promoter
M2tri Fmu	agcttTCCACATATCAGGTTATTTCCACATATCAGGTTATTTCCACATATCAGGTTAg	Cloning oligo for mutated *PIF4* promoter
M2tri Rmu	tcgacTAACCTGATATGTGGAAATAACCTGATATGTGGAAATAACCTGATATGTGGAa	Cloning oligo for mutated *PIF4* promoter
**Gene cloning**		
PIF4 F	CCGCGTCCTGTATTTGGCTA	Cloning primer for *PIF4*
PIF4 R	GTGGTCCAAACGAGAACCGT	Cloning primer for *PIF4*
pPIF4 F	CCGCGTCCTGTATTTGGCTA	Cloning primer for *PIF4* promoter
pPIF4 R	GTCAGATCTCTGGAGACATTTCA	Cloning primer for *PIF4* promoter

pCCA1 F	TCATGCATGGTTAGCTTAGCTTA	Cloning primer for *CCA1*
CCA1 F	ATGGAGACAAATTCGTCTGGAG	Cloning primer for *CCA1*
CCA1 NR	ATTCATGGAGGATGCAGCAGAGA	Cloning primer for *CCA1* N terminus
CCA1 CF	ATGGAGATGGCCAATGAAGATCGA	Cloning primer for *CCA1* C terminus
CCA1 R	TGTGGAAGCTTGAGTTTCCAA	Cloning primer for *CCA1*
pLHY F	GGTTAGTAACAAAAACTGGTCTATT	Cloning primer for *LHY*
LHY F	ATGGATACTAATACATCTGGAGAAG	Cloning primer for *LHY*
LHY NR	TAAGGGATACTTGTTCACAG	Cloning primer for *LHY* N terminus
LHY CF	ATGTTGAATCAGGCGTTCTTGGA	Cloning primer for *LHY* C terminus
LHY R	TGTAGAAGCTTCTCCTTCCAAT	Cloning primer for *LHY*
SHB1 F	ATGAGGTTTGGGAAAGAGTT	Cloning primer for *SHB1*
SHB1 NR	GCTTGTTAGTTGATCTCCCAAGA	Cloning primer for *SHB1* N terminus
SHB1 CF	ATGGGCACTGAACTTGGTTATA	Cloning primer for *SHB1* C terminus
SHB1 R	ATTGTTATGATGATCTCCATCTT	Cloning primer for *SHB1*
4M1m-1 F	TAATTGCTTTATGCTCCTGTCACTTTCT	Forward primer for M1 box base substitution mutation
4M1m R	CAGCGAGGGAGATAATGATTCTCT	Reverse primer for M1 box base substitution and deletion mutation
4M1m-2 F	TATTATGCTCCTGTCACTTTCTGTCTGT	Forward primer for M1 box deletion mutation
**Genotyping**		
X5	GAAGATACGGGTTTTGCAT	Genotyping primer for *shb1-D*
701	GGGAAGCTTGGATGTCTTGAA	Genotyping primer for *shb1-D*
JL202	GGGAAGCTTGGATGTCTTGAA	T-DNA left border primer for *shb1-D*
SHB1 LP	TTGTAGTTTTGCACATGGACG	Genotyping primer for *shb1*
SHB1 RP	TCCCCAGTCAAGAACAATGTC	Genotyping primer for *shb1*
PIF4 LP	ATGGAACACCAAGGTTGGAGT	*PIF4* 5’ primer
PIF4 RP	GTGGTCCAAACGAGAACCGT	*PIF4* 3’ primer

PIF4 Lb	AACCTGCGTGCAATCCATCT	T-DNA left border primer for *pif4*
LBb1.3	ATTTTGCCGATTTCGGAAC	T-DNA left border primer
CCA1 F1	AAAGCTGAATCATCTCTTCAGC CACTAGT	Genotyping primer for *cca1*
CCA1 R1	GCTTGCGTTTGATGTCTCT	Genotyping primer for *cca1*
LHY F1	CTTACCAACGAAAGTAAGTCTA AGAAAGC	Genotyping primer for *lhy*
LHY R1	AAAGATTGGAGAAGCAAACTAC TAACACT	Genotyping primer for *lhy*
LHY R	AACCTGACATGACCAAAGAAAT GTTCGGA	Genotyping primer for *lhy*
JL270LB	TTTCTCCATATTGACCATCATAC TCATTG	T-DNA left border primer for *cca1* and *lhy*
